# Alienation in the Teaching Hospital: How Physician Non-Greeting Behaviour Impacts Medical Students’ Learning and Professional Identity Formation

**DOI:** 10.5334/pme.1185

**Published:** 2024-04-18

**Authors:** Eivind Alexander Valestrand, Beth Whelan, Knut Eirik Ringheim Eliassen, Edvin Schei

**Affiliations:** 1 Center for Medical Education, Faculty of Medicine, University of Bergen, Norway; 2 Department of Global Public Health and Primary Care, Faculty of Medicine, University of Bergen, Norway; 3 Student Health and Wellness Center, Memorial University, St. John’s, NL, Canada

## Abstract

**Introduction:**

Clinical workplaces offer unrivalled learning opportunities if students get pedagogic and affective support that enables them to confidently participate and learn from clinical activities. If physicians do not greet new students, the learners are deprived of signals of social respect and inclusion. This study explored how physicians’ non-greeting behaviour may impact medical students’ participation, learning, and professional identity formation in clinical placements.

**Methods:**

We analysed 16 senior Norwegian medical students’ accounts of non-greeting behaviours among their physician supervisors in a reflexive thematic analysis of focus group interview data.

**Results:**

The main themes were: A) *Descriptions of non-greeting*. Not being met with conduct signalling rapport, such as eye contact, saying hello, using names, or introducing students at the workplace, was perceived as non-greeting, and occurred across clinical learning contexts. B) *Effects on workplace integration*. Non-greeting was experienced as a rejection that hurt students’ social confidence, created distance from the physician group, and could cause avoidance of certain workplace activities or specific medical specialties. C) *Impact on learning*. Non-greeting triggered avoidance and passivity, reluctance to ask questions or seek help or feedback, and doubts about their suitability for a medical career.

**Conclusion:**

Medical students’ accounts of being ignored or treated with disdain by physician superiors upon entering the workplace suggest that unintended depersonalising behaviour is ingrained in medical culture. Interaction rituals like brief eye contact, a nod, a “hello”, or use of the student’s name, can provide essential affective support that helps medical students thrive and learn in the clinic.

## Introduction

Medical students cannot achieve adequate learning and professional identity formation during clinical placements unless they get practical, pedagogical, and affective support from physician supervisors [1]. Physician support can facilitate learning by empowering students to develop the agency to take risks and responsibility giving them access to real patient learning, and treating them as legitimate members of the physicians’ community of practice [1,2,3]. However, clinical learning environments, where patient care has top priority, may provide suboptimal support for learning [4,5]. Students can feel burdensome, belittled, and mistreated in non-supportive clinical environments [6,7]. Despite increased interest in the psychological and relational aspects of workplace learning, we find a scarcity of research into how relational behaviour and interaction rituals, such as greetings or lack thereof, impact medical learners’ socialisation, motivation, and learning in the workplace. Since greeting behaviour is vital in all cultures and plays an essential function in establishing rapport, trust, and rank in unfamiliar social settings [8], addressing physician non-greeting and its consequences for learning has potential for improving the quality of medical students’ clinical placements.

Studies in social anthropology [8], sociology [9], and psychology [10], show that greetings and small talk are vital in facilitating ‘detailed management of interpersonal relationships during the psychologically crucial margins of interaction’ (p. 217) [8]. Relationship-building rituals are particularly important when people do not know each other well or have unequal social standing [11], which is the case for medical students entering new clinical workplaces. The more powerful participant, such as a physician supervisor, may greet first to give the other ‘safe conduct to enter his territory without making him suffer a counter display of hostility’ (p. 226) [8]. Depending on the environment, different signifiers such as a nod, a handshake, an embrace, a smile, or ‘empty’ phrases like ‘how are you?’, are used to greet [12]. Names are typically exchanged if the people have not met before and circumstances allow. Greetings communicate information about the speakers’ identities, attitudes, and relative rank, thus shaping the nature of their contact. This is called the ‘phatic’ function of interaction rituals, which serves to establish or maintain a social relationship through non-verbal and verbal communication [8].

When established members of a work environment skip greetings, it deprives newcomers of the phatic signals that are needed to establish psychologically safe relationships with superiors, colleagues, and other staff [13]. Non-greeting can be verbal or non-verbal behaviour that fails to provide a newcomer with the conventionally expected signs of being socially respected and included. Non-greeting is likely to be interpreted by the newcomer as a rejection, a hostile signal that one is not wanted, or is unimportant, in the community represented by the host. What will be perceived as a non-greeting depends on the context, as conventional introductory behaviour varies between cultures and communities [14]. The consequent lack of relatedness, support, and autonomy, and missed opportunities to ask, participate and learn through trust-based interaction, may trigger defence mechanisms and make students experience themselves as inauthentic, not enacting their true selves [15,16].

In medical education, neglect has been identified as harming students’ learning and well-being, through behaviour that borders on mistreatment [17,18]. Not greeting students can be understood as a specific neglectful behaviour, that may make students preoccupied with conforming to the perceived expectations of others [19]. This can in turn increase anxiety, harm student performance, and thwart learning [20]. A study of nursing students revealed that not being greeted by senior staff during clinical placements could alienate students from their professional role [21].

This study explored medical students’ experiences of physician supervisors’ greeting behaviour during clinical placements. We sought to answer: What are the impacts of physicians’ non-greeting on students’ participation, learning, and professional identity formation?

## Material and methods

### Context

This study was conducted at a single university in Norway, in which undergraduate medical education lasts six years, with at least 24 weeks of clinical placement during the last three years. Students typically rotate to new wards every two to five days, which frequently exposes them to new workplace environments where physicians and other staff are unknown. As a group, the students in this study had experienced clinical placements in four different hospitals of varying sizes. In Norway, physicians in teaching hospitals are formally required to support and supervise medical students, but are often unaware of this duty, and may not identify as supervisors. Most have no formal pedagogical training. Students are usually not assigned a personal supervisor for the placement.

Social anthropology has established that all cultures use phatic talk or gestures, and that lack of it is usually deemed impolite by those on the receiving end [22,23]. There are relatively subtle displays of hierarchy in Norwegian workplaces, and the social norm is that those who are familiar with the workplace should greet newcomers. A standard greeting of a new person in a workplace would entail a firm handshake or arm gesture, eye contact, sometimes accompanied by a smile, and stating one’s name [24].

### Study design

This study was explorative, qualitative, and placed within the socio-constructivist paradigm [25,26]. This paradigm was chosen due the topic in question being socially situated. We explored data from focus group interviews initially conducted to investigate Norwegian medical students’ experiences of shame in the learning environment. Focus group interviews were chosen as they are suitable for exploratory research, as sharing stories can stimulate discussion and normalize individual experiences [27,28].

This study is a secondary analysis. The first analysis focused on students’ narratives of shame in clinical placements [7], and identified that not being greeted by physician supervisors caused medical students to experience shame. The current study was designed to explore the concept of non-greeting more fully by utilizing a richness in the material that was not made use of in the first analysis. After an evaluation of the dataset, we considered its quality, sufficiency, and relevance suitable for a secondary analysis [29]. We applied a new research question, theoretical framework, and methodology to ensure this study was distinct from the first analysis, while targeting an overlooked and significant knowledge gap in the medical education literature [30]. Co-authors EAV and KEE were brought into the project to ensure the dataset was also analyzed by researchers who had not previously interacted with the data.

Participants were recruited using a snowball approach among fifth- and sixth-year medical students. Class representatives were informed about the study and forwarded an invitation to classmates through direct contact and social media. Interested students contacted BW by email to confirm participation. We offered no incentives apart from tea, coffee, and snacks.

Three 90-minute focus-group interviews were conducted in English with a total of 16 participants (12 female, 4 male, ages 24–33). The gender balance among participants was representative of the student population (71% female, 29% male). Each group consisted of four students from one class, with two groups of fifth-year and one of sixth-year students. Participants were prompted by email to think about medical education experiences that they associated with feelings of shame. Before starting the interview, we provided informed consent and confidentiality forms. BW and ES moderated the focus groups using a semi-structured interview guide (see Supplementary file 1: Interview guide).

During the interviews, the concept of not being greeted arose spontaneously. Interviewers followed up on individuals’ stories, inviting students to describe, reflect on, and discuss their experiences and the perceived consequences. Interviews were audio-recorded and transcribed. Participants could withdraw from the study at any point before publication.

### Ethical considerations

Data was anonymised, and person-identifying information has been removed from the transcripts. Participants are given fictitious names that indicate gender while ensuring they are unidentifiable in this article. The focus group (FG) number is specified for each quote.

### Data analysis

We completed a reflexive thematic analysis investigating the transcribed interviews for elements related to physician-student greeting behaviour [31,32,33]. This analysis is suitable for exploring patterns in individual experiences through a thoughtful engagement with the data and the analytical process. Before analysis started, transcripts were cleaned of all coding from the first analysis of the data. To start, all authors familiarised themselves with and discussed the complete dataset in a meeting. Three authors (EAV, BW, KEE) then separately reread the material and generated initial codes before individually collating codes into potential themes based on shared patterns of meaning. Then, the research team got together to share and discuss what they had done. Over multiple meetings we reviewed themes before deciding which best answered our research question. These themes were then defined and given clear names. The fourth author (ES) took on a central role of questioning assumptions and interpretations throughout the analytic process. We completed the last parts of the analysis as we wrote the manuscript, including the selection of quotes, a final analysis of the data, and relating our findings to the theoretical literature.

### Reflexivity

Reflexivity relates to positionality, to how researchers frame their thinking and bring themselves into the research process [34]. Analysis benefited from investigator triangulation achieved by harnessing BW’ and ES’s insights from previous data analysis and the fresh perspectives of EAV and KEE. Throughout the analysis, we identified and discussed our assumptions. For positionality, the research group represents a variety of competencies and lenses for observation and analysis, including medical (EAV, KEE, ES) and psychological (BW) training, clinical supervision (KEE, ES), and educational research (all authors). Our research interests include self-conscious emotions, medical professionalism, leadership, communication skills, and professional identity formation.

Our understanding of a greeting is that it entails a non-verbal or verbal action towards another person, signalling acceptance or rejection of them. What is a proper greeting will often change over time, from formal and thorough at the beginning to casual and potentially subtle between friends and close colleagues. There is no single way of greeting, and there may be differences between groups in what is deemed a customary greeting. For example, different generations may adhere to certain conventions, such as older people often using surnames, whereas younger people prefer using given names. In the clinical learning environment, we believe responsibility lies with the established professionals to be welcoming and inclusive towards newcomers and learners. The newcomer’s responsibility is to be humble, polite, curious, and open to being involved in the environment as it is.

## Results

In the following, we present the three main themes from our analysis; 1) *Descriptions of non-greeting*, i.e. the behaviour students experienced as a non-greeting; 2) *Effects on workplace integration*, i.e. the impact not being greeted could have on a social level; and 3) *Impact on learning*, i.e. the impact not being greeted could have on a personal level. Themes 2 and 3 are intrinsically linked and have some overlap.

### Descriptions of non-greeting

The participants described a range of physician behaviours as non-greeting, for example, avoiding eye contact, not saying hello, not using the students’ names, or not initiating contact with a designated student despite supervisory responsibilities. Participants recalled such behaviour across learning contexts, including upon arrival on unfamiliar wards, morning meetings, rounds, clinical skills training, and during patient visits. Non-greeting could set a precedent for future interaction between a student and an individual doctor and other doctors in the same clinical team.

‘*We went to the morning meeting 10 minutes before and people kept coming in and there were no chairs, so we stood up against the walls. And the doctors were like, who are you? … Then during the meeting, we were not addressed one single time as students. The meeting starts, it develops, and it ends and then everyone stood up and left.’ (Iris, FG2)*

Lack of seemingly banal interactions, like small talk, could make it more difficult for participants to engage in subsequent professional conversations. Other examples showed physicians treating students like underlings, not bothering to learn their names, and ordering them around. Three participants had similar experiences of being objectified as ‘the student’:

‘*And often they haven’t even heard what you’re called, they just call you student:*‘

*The student can listen to your heart; the student can feel your belly’.’ (Olivia, FG3)*

‘*May the student touch your toe, please.’ (Natalia, FG3)*

‘*Can the student leave the room. That’s nice!’ (Michael, FG3)*

Several participants reported that doctors’ body language was an important signifier alerting them to whether they could trust the relationship to be welcoming and supportive. They recalled doctors who would not even provide eye contact when greeting them, interpreting this as if eye contact entailed unwanted responsibility for the student. Other stories were about supervisors who would tell students they had no tasks for them to take part in, while having their backs turned towards them.

‘*I think it’s about body language … If someone says “Leonard, you should be with this doctor today” and [the doctor] just walks away, it’s very hard. You need to look someone in the eyes to say hello to them.’ (Leonard, FG3)*

Participants described how attempts to initiate contact with physicians were ignored or even met with sarcasm or hostility. In the following example, a participant was attending a morning meeting for the first time and, without being greeted, was told to sit in a specific place. After a few minutes this happened:

‘*One of the surgeons said: “You’re in the way. Move!” And I thought: “You told me to sit here!” I didn’t say that, but that’s what I thought. But ok, so I moved, and they brought the screen down, did the whole meeting and didn’t say anything except: “Move, you’re in the way.’ (Dennis, FG1)*

### Effects on workplace integration

Absence of greeting behaviour was interpreted as hostile phatic signals, a perceived rejection that triggered defence mechanisms and left students frustrated, passive, and feeling like an unwelcome burden in the professional community.

‘*If you haven’t said hi to the doctor and you haven’t said hi to the patient, then you are no one, you’re just a student, or a guy in a white coat.’ (Leonard, FG3)*

Instances of not being greeted led participants to avoid specific settings in the workplace and even narrow their career plans by ruling out certain medical specialties. More generally, lack of social recognition increased student reluctance to get involved in the clinical work and engage with patients and personnel. Some lost confidence in their suitability for a medical career. When participants were not supported in becoming an integral part of the work environment, some became overly preoccupied with the complexity of social interactions. The following quote from a participant talking angrily about his clinical placement exemplifies this:

‘*After a while I’ve been really scared of surgery. They kind of let me in and exploited me a bit. After half an hour they said look at this, that is a ligament and I said oh, thanks for teaching me anything [sarcasm]. Because you’re so busy with the tension in the room … especially when the doctors don’t give you any attention. I find it very hard to learn anything. There’s been so many days I have been at school for six hours and then I go home, and I think I have not learned anything today.’ (Alice, FG1)*

Many participants reported that non-greetings made them feel less safe in the environment and uncertain about how they could contribute. Some experienced a lack of agency in clinical situations, feeling peripheral to the clinical encounter, as they had been given no opportunity to be themselves as members of the physicians’ community. This participant emphasised the sense of not belonging when introductions were not made:

‘*A lot of the time you just stand in the background. I stand further back when I haven’t introduced myself because I’m subconsciously not in the situation. You don’t feel like you belong as much when you haven’t had a chance to say who you are.’ (Michael, FG3)*

When participants were not addressed by name, many did not feel seen as individuals. It distanced them from the physicians, as they felt that the physicians did not care about them and that they, as students, were part of an outsider group with no purpose in the clinic. When participants’ names were not used it was harder to establish and sustain a functioning supervisor relationship.

‘*You know you’re going to be with this doctor for the whole day. … You are following them, and they don’t say hi to you. You know their name because you know who they are, but they don’t know your name. It’s really hard to be with a person without introducing yourself first.’ (Leonard, FG3)*

Many participants had come to terms with not being greeted, saw it as part of the medical culture, and had not consciously reflected on or talked about these experiences with other students before being invited to the focus group interview. They had never openly criticised superiors’ behaviour and had informed neither faculty nor hospital administration about physician non-greetings or lack of politeness. The professional hierarchy of the hospital setting made participants feel powerless and compliant.

‘*… when [the senior surgeon] says it, maybe it is supposed to be like that. I’m just a medical student.’ (Dennis, FG1)*

### Impact on learning

Participants frequently mentioned how non-greetings made them focus on themselves and their difficult emotions rather than engaging in a clinical encounter or observing the technical details of a procedure. Not having been greeted by a supervisor could distract them from patients and compel them to reflect on themselves and doubt their place in medicine, rather than concentrate on what they could learn and how they could develop as doctors. Participants expressed that being greeted helped them have confidence and agency as fledgling colleagues in the clinical environment. A greeting could signal that someone was looking out for them and could generate a safe space from which they could explore medicine, their professional identity, and the complexities of the hospital workplace. Conversely, a lack of greeting could impact participants’ ability to take initiative and try things for the first time.

‘*It’s much harder to launch yourself into something new if you don’t know if anyone is there to catch you. Then you don’t have … the greeting, the acknowledgement that you’re a student.’ (Olivia, FG3)*

Lack of greeting could trigger disorientation, impact participants’ understanding of their professional identity in the clinical context, and harm their motivation to explore and learn medicine.

‘*You just feel stupid kind of. … I just didn’t understand why I was even there, is it just to see how a ward works? Should I be trying to learn something, should I ask questions about subjects? I was very confused to be honest.’ (Iris, FG2)*

Non-greetings made participants feel disrespected and disdained, making them unsure about the purpose and meaning of being in clinical placement.

‘*It felt awful, they didn’t acknowledge me, and I thought maybe I should just leave. I felt like nothing, like a waste of space.’ (Dennis, FG1)*

If clinical supervisors did not initiate conversations and remained silent in the presence of students, the participants described a feeling of being invisible. They dealt with this by becoming passive, not initiating talk or asking questions, or just tagging along without knowing to what end.

‘*He wasn’t addressing me at all, so I thought I’m just going to shadow you or whatever, and then suddenly we were in the men’s room.’ (Karen, FG2)*

Participants who had not been greeted and just followed behind the doctor were also frequently not introduced to the patients during clinical rounds. This could prompt uncertainty and make students fret about their role and position in the encounter, rather than focus on the patient and clinical learning.

‘*They just start talking to the patient and then you think I didn’t have a chance to introduce myself, so the patient doesn’t know who I am. You think ‘should I interrupt the conversation between the doctor and the patient and say hi I’m Natalia, I have a purpose, I’m a student, and I’m here to learn?’.’ (Natalia, FG3)*

While some participants attributed their unpleasant experiences to flaws in the medical culture at large, and tried not to take it personally, it was common that non-greetings generated uncertainty about their current identity and made them question whether they were personally fit to become a doctor. The experience of not being greeted could make them ruminate on not being adequate, having a character flaw, or having displayed inappropriate behaviour.

‘*Maybe it’s because I did something wrong.’ (Dennis, FG1)*

## Discussion

This study reveals how physicians’ non-greetings and other omissions of social stewardship, such as not presenting medical students to patients or not using their names, can be perceived as social rejections that harm medical students’ learning and professional identity formation. As a result of supervisors’ non-greeting behaviours, participants reported feeling embarrassment, confusion, impaired agency, uselessness, reduced motivation for learning in the workplace, and disidentification with the medical profession.

The descriptions of non-greetings varied in severity, from clinicians’ ignoring of students, to being rude or showing direct or indirect hostility. Such incivility and neglectful behaviour, with its spectrum of dismissive conduct, is likely to be under-reported in medical education [18,35]. The specific descriptions given by students in this paper can help physicians and educators understand how unintended incivility might be perceived from the student’s perspective.

Furthermore, we believe the word ‘phatic’ deserves a place in the medical education literature. It is a well-described theoretical concept that directs attention to and explains how seemingly trivial social behaviour is essential for building trust and predictability at the margins of social interactions, when insiders and newcomers, seniors and juniors, physicians and patients, meet and establish relationships [8]. Our findings show that conventional displays of politeness and welcoming when students arrive at a clinical workplace can be crucial for the students’ learning, professional identity, and career path. The phatic function of initial social interactions lies not in the intention of the sender but in the sub-conscious interpretation of the receiver, who in such situations looks for assurance that this is a safe place while seeking to gauge ‘who are you’ and ‘who am I to you’ [36]. The resulting social connection can create agency, motivation, and potential opportunities for shared reflection between students and supervisors about what the students experience during patient encounters and other clinical activities. Being recognised and allowed to form relationships with physician supervisors can make students feel like future colleagues, who belong and matter in the community [37,38,39]. In contrast, overlooking or ignoring a student can stir emotions that negatively impact learning and impede their integration into the community of physicians [3,40].

This study highlights students’ vulnerability during clinical learning, and their need for social signals that allow them to feel welcomed and involved in the workplace. Seemingly innocent aspects of physician behaviour can be detrimental to medical students’ learning and professional identity formation. When students do not feel welcome, it can erode their sense of agency and motivation. Students in our study became focused on self-monitoring rather than on what they could learn. Lack of collegiality and impoverished sense of belonging or mattering have been related to increased burnout in medical students [41,42]. In contrast, connecting to colleagues, patients, and the profession itself may be vital in strengthening medical students’ resilience [43]. Without the implicit promise of affiliation mediated by a greeting, it can be difficult for students to be authentic and autonomous, thus hampering learning and professional identity formation [20,44]. In contrast, if students experience psychological safety, they can be freed from constant self-monitoring, be present in the moment, and engage with the learning tasks at hand [45,46].

The experience of repeatedly being called ‘student’ rather than by name was particularly difficult, as it ignored students’ individuality and made them feel invisible and irrelevant. The importance of using students’ names needs to be emphasised. Hearing others call them by name will uniquely activate their brains in regions related to making judgements about themselves and their personal qualities [47]. It can make them feel called upon as individuals, strengthening their sense of belonging and mattering [48].

Most participants had not talked about their non-greeting experiences before. This may stem from an impulse to conceal disturbing experiences of vulnerability and perceived uselessness. The lack of sharing may also signal that students trust the existing culture and assume that there are legitimate reasons for the way it is, and if they cannot tolerate it, there may be something wrong with *them* rather than the culture. Such cognitive mechanisms can make students accept a suboptimal learning environment, and even believe it is necessary and normal [49]. This process is likely part of the reason such flaws in culture persist over time, as new members in the community unconsciously, and unintendedly, learn from their superiors to accept that certain ways of being are expected within medical culture and are integral to being a doctor [50]. As participants shared their experiences during the interviews, they saw that non-greetings were common, reprehensible, and challenging even to others. This helped them appreciate that the problem did not lie within themselves and made it possible to reinterpret their experiences.

Our findings reveal that some physicians display a lack of courtesy, care, and professional ethics. These moral aspects of being should ideally be interwoven with the physicians’ own professional identity and are social skills that they most likely manage in other domains of life. One explanatory mechanism may be that clinicians often do not identify as educators [51], are unaware of the magnitude of their power to help or harm students [52], and underestimate the importance of greeting in the workplace [53]. Another cause of doctors’ blindness to their potential impact on students’ workplace learning may be the current endemic of physician burnout, which can lead to emotional exhaustion and objectification of others [54]. If so, insensitivity, lack of empathy, and negative attitudes are defence mechanisms not aiming to hurt students, but to protect the physicians themselves. The ‘imposter syndrome’, a commonly experienced tendency toward self-doubt, may make doctors cautious of engaging with students, and prompt avoidance or aggression towards them [55]. This may constitute a ‘prosociality paradox’ in which clinicians avoid greeting students believing they do not have the competence to help them, failing to recognise that students seek acceptance, warmth, and safety, as well as medical expertise [56]. Furthermore, physicians’ task-oriented focus on ‘producing’ efficient patient care as the overarching goal of the system they work within, can cause them to perceive students as potential obstructions [57,58], and explain why they sometimes treat students as objects. This resonates with previous research [59]. These processes may contribute to ingraining depersonalising behaviours into the medical culture, making it a common way of behaving as a doctor even though it has harmful effects on students, patients, and the doctors themselves.

In this study, we see how frequent rotations to new wards and clinical departments can force medical students to play the role of a newcomer again and again. Being met repeatedly with inhospitable behaviour from unfamiliar physicians and other health professionals can facilitate a recurring and cumulating sense of disempowerment. Structural arrangements allowing students to spend extended time on each ward may help them establish relationships, integrate into the community of practice, allow familiarity and curiosity to evolve, and strengthen their possibility for achieving the intended learning outcomes of the clinical placement [60]. Still, regardless of the length of a placement, medical students learning will benefit from being welcomed.

The study has some limitations. First, the data come from focus-group interviews exploring shame in medical education, which may have influenced the examples discussed in the groups. The emotion-oriented framing may have functioned as an opener for students to access troublesome experiences that they otherwise would not have reflected on, due to both their uncomfortable and seemingly trivial nature. Still, we might have had richer material if the interviews had been directed toward non-greeting events. Secondly, we interviewed medical students from one medical school, in which students frequently rotate between wards during clinical placements. While this may limit the transferability of our results, it may also have revealed the phenomenon and the effects of non-greeting more readily. We believe the findings of this study are of general relevance, as the need for being welcomed and greeted is universal, and highly important at the outset of any clinical placement.

Future research should explore how clinicians perceive the importance of greeting in medical workplaces, and their experiences of receiving new students in the clinic. It is also important to gain more knowledge regarding the experience of being greeted or not, depending on context, gender, ethnicity, or being a representative of other minoritized groups. Faculty development initiatives to improve clinicians’ greeting behaviour towards students should be evaluated.

### Implications

Educators and clinicians need to understand the harmful consequences of non-greeting and other phatic omissions for students’ clinical workplace integration, learning of skills and knowledge, and professional identity formation. Medical students need pedagogic support that can help the doctors of tomorrow avoid adapting to and propagating a dysfunctional culture.

Improving physicians’ greeting behaviours should not be too challenging, since it only demands a level of phatic awareness that they most likely have in other social settings. By behaving courteously in their everyday workplace interactions, medical staff can provide the necessary impetus for change of behaviour patterns [61,62], thereby possibly nudging medical workplace behaviour from impersonal to more person-centred, to the benefit of students, clinicians, and – hopefully – patients.

## Conclusion

Our study shows that physicians’ non-greeting behaviour in the clinical workplace can create psychologically unsafe learning environments where medical students do not feel welcome and secure, thereby curtailing agency, impairing social integration, harming professional identity formation, and alienating newcomers from the medical profession. Our findings indicate that unintended depersonalising behaviour may be an integrated facet of medical culture itself, a way of manifesting oneself as ‘a good doctor’, in consonance with the profession’s tradition for forefronting efficiency, expediency, and objective facts. However, our student informants also convey that seemingly small acts of greeting, such as a nod, a ‘hello’ from physicians and other staff, or use of the student’s name, have phatic functions that are essential for social functioning and powerful catalysts of the agency and perceived support that students need to fully profit from the huge learning potential of clinical placements.

## Additional File

The additional file for this article can be found as follows:

10.5334/pme.1185.s1 Supplementary file 1. Interview guide.
