# Supplementary material for: Alienation in the Teaching Hospital: How Physician Non-Greeting Behaviour Impacts Medical Students’ Learning and Professional Identity Formation
Source: Perspect Med Educ. 2024 Apr 18;13(1):239–49. doi: 10.5334/pme.1185 (PMC11025575; doi:10.5334/pme.1185)
Supplement: Supplementary file 1. — Interview guide. [file pme-13-1-1185-s1.pdf]

Introduction:
Prior to the first focus group meeting, students will be asked to write about an experience of their choice, in which they experienced shame in the context of medical education. We will let students know that we will be discussing these stories in the context of the focus groups, therefore we suggest they write about something they would be comfortable sharing with the group. These reflections will assist in guiding the discussion during the focus groups, as we will ask students to be prepared to share their stories within the context of the focus group.

We will also inform students that reflections will be collected at the end of the focus group, and they will be anonymized to protect the identity of the students.

Instructions: Sit by yourself and reflect upon a clinical situation(s) in which you felt flawed, deficient, or unworthy, or a situation which you experienced as shameful. Write about this event and describe what happened. It would be helpful for you to include what happened in your body, and any thoughts and emotions that were present during this experience.

Semi-Structured Interview:

You have all taken some time prior to this meeting to write about an experience in which you felt flawed, deficient, or unworthy, or a situation which you experienced as shameful.
During this focus group we would like to take this opportunity to discuss these stories in a more in-depth way.

To facilitate openness the facilitator may start with a brief vignette describing a time when he/she experienced shame (as a means of normalizing the experience).

We hope to have an open discuss about this topic, with questions and comments arising spontaneously from the discussion.

Additional themes/questions that may be used to facilitate discussion are listed below.

In this situation, why did you feel deficient, flawed, or unworthy?
- What specifically did you feel was deficient or flawed about yourself in the context of the described experience?
- How did you physically feel as a result of feeling flawed/deficient/unworthy? In other words, what physical reactions did you experience?
- What actions did you take or want to take as a result of feeling flawed/deficient/ unworthy?
- How long did the overall feeling of being deficient, flawed, or unworthy last?
- What were the outcomes or effects of feeling flawed/deficient/unworthy?
- Is there anything else that is important about this situation that you would like to tell me?

Interview questions about general shame experiences:

Interviewer: "Having introduced shame as a term, I'd like to talk a little more about any other experiences you may have had with shame."

- Other than the situation you described, what other shame reactions have you experienced as a medical learner, if any?
- What other types of events, actions, or experiences cause you to feel shame?
- How often do you think you feel shame? In other words, how often do you feel deficient/unworthy/flawed?
- What, if anything, do you do if/when you experience shame? In other words, what actions do you take (or want to take) in response to your feelings?
- What effect(s) and/or outcomes(s) do these shame experiences have on you?
  - o How do these feelings change your approach to your education? To your approach to patient care? To your approach to the learning environment?
  - o How do these feelings change the way you view yourself as a physician in-training, if at all?
  - o How do these feelings change the way you view yourself as a whole person, if at all? In other words, how have these feelings impacted the way you view yourself outside of the hospital?

Interview questions about identity—goal congruence, attributions, and self-evaluation:

Interviewer: "I would like to better understand the thought processes and influences that may have led to the emotions you experienced as a result of the situation you described."

- What were your goals and expectations for yourself in the situation you described? In other words, how would your "ideal" self have acted?
- From where did these goals and standards arise? In other words, who or what influences the goals and standards you've set for yourself?
  - o Where did they come from?
  - o When did they arise?
  - o How reasonable and/or achievable are these standards?
  - o Do you hold other people to the same standard? Why or why not?
- How often do you evaluate yourself as a resident in general?
  - o What influences the way you evaluate yourself? In other words, what influences whether you are hard on yourself or kind to yourself?
- Are there any specific environmental influences, either at or outside of work, that influence your tendency to feel shame or directly cause you to feel shame?
  - o If yes, can you describe those influences to me?
  - o If no, can you think of any environmental influences that may cause others (i.e., other students, residents, or others) to feel shame?
